# Dementia trials and dementia tribulations: methodological and analytical challenges in dementia research

**DOI:** 10.1186/s13195-015-0113-6

**Published:** 2015-03-18

**Authors:** Craig W Ritchie, Graciela Muniz Terrera, Terence J Quinn

**Affiliations:** Department of Psychiatry, Centre for Clinical Brain Sciences, University of Edinburgh, Edinburgh, EH10 5HF UK; MRC for Lifelong Health and Ageing, University College London, London, WC1B 5JU UK; Department of Academic Geriatric Medicine, Institute of Cardiovascular and Medical Sciences, University of Glasgow, Glasgow Royal Infirmary, Glasgow, G4 0SF UK

## Abstract

Dementia is a substantial and increasing public health concern. Despite decades of research, a cure or effective preventative treatment for dementia remains elusive. We offer critical review of contemporary dementia research and discuss potential reasons why progress in the field has not been as rapid as in other disciplines. We adopt a broad approach in keeping with the broad nature of the topic. We cover the difficulties inherent in studying dementia from 'bench' to 'bedside' to 'population'. We make particular reference to issues of operationalisation of the dementia syndrome and our evolving understanding of dementia as a research 'outcome'. We discuss contemporary 'hot topics' in dementia research methodology focussing on dementia models, pre-dementia states and biomarkers. Recognising the importance of prospective epidemiological cohorts and large-scale clinical trials we pay particular attention to these approaches and the challenges of generating results that have 'real world' external validity. Based on our thoughts we end with suggestions for future dementia research. Our review is designed to be critical but not unnecessarily negative. There is reason for cautious optimism in dementia research. The recent G8 summit on dementia and subsequent establishment of the World Dementia Council are examples of initiatives that reflect societal and political will to increase research efforts in dementia.

## Introduction

The scientific and lay press frequently remind us of the changing global demographic. Increasing longevity should be celebrated as a medical and public health success, but with increasing age comes (currently) increasing prevalence of age-associated diseases, including the dementias. The evidence-based medicine movement has facilitated major advances in our understanding and treatment of disease, but progress has not been equally shared amongst diseases. While cardiovascular disease research has yielded a wealth of effective primary and secondary preventative treatments, dementia remains less well understood with a paucity of effective treatments. This inequality in treatment options is reflected by current research funding; research support for dementia is modest compared with that for cardiovascular disease or cancer [1].

In this review we consider some of the limitations and challenges of researching dementia. It would not be possible to comprehensively describe the entire dementia research field in a single review and we have chosen to focus on those areas we feel are most pertinent to contemporary clinicians and researchers. Recognising the difficulty of studying a condition where diagnosis is 'clinical', we begin by describing how we operationalise the dementia syndrome. We then review three 'hot topics' in dementia research: (i) dementia models, (ii) biomarkers and (iii) 'pre-dementia' states. We conclude with discussion of large-scale studies, both observational cohorts and interventional trials.

## Operationalising dementia

### What is dementia? A researcher’s perspective

A fundamental problem in dementia research has been defining exactly what we are researching. Arguably, the complex and reductionist taxonomy of dementia theory has led to compartmentalised thinking and research. As a research 'outcome' dementia can be operationalised at various levels. We have developed a nosological system wherein dementia can be diagnosed as a syndrome and further classified by presumed underlying disease (for example Alzheimer’s) and this classification can be subtyped again (Alzheimer’s variants). Classification by the clinical symptomatology, the neurohistopathology, characteristics of the patient group ('pre-senile' dementia) or site of predominant anatomical change ('subcortical') have all been employed [2]. While many of these classifications are now obsolete, new technologies, particularly dementia 'biomarkers', may foster a plethora of new research terminologies and labels [3].

Dementia remains a clinical diagnosis and this diagnosis is usually made using standardised classification systems such as the American Psychiatric Association Diagnostic and Statistical Manual of Mental Disorders (DSM) or the World Health Organization International Classification of Disease (ICD) [4,5]. These lexicons bring a degree of clarity but it is worth noting that there are differences in their approaches, and studies comparing contemporaneous assessment with ICD and DSM reveal potential disagreements in classification [6]. Neither system is superior to the other and both have been criticised for their reliance on memory impairment to make a diagnosis and their focus on defining exclusive disease subtypes and their use of criteria designed to capture disease only once it is clinically obvious and other conditions have been fully excluded [6].

A revision of DSM (DSM-V) was published in May 2013 and revision of ICD (ICD-II) is anticipated [4]. DSM-V has moved from a focus on memory and has redefined 'dementia' as 'neurocognitive disorder' with qualifiers of 'major' or 'minor' disorder. Major neurocognitive disorder is not synonymous with 'dementia' as previously described and we will have to be mindful of this when interpreting and comparing data from studies that use different iterations of DSM. Classification system content continues to lag behind improvements in our understanding of natural history and pathogenesis. In the rapidly evolving landscape of contemporary dementia research, more frequent revision of diagnostic classification may become necessary.

**Figure 1:**
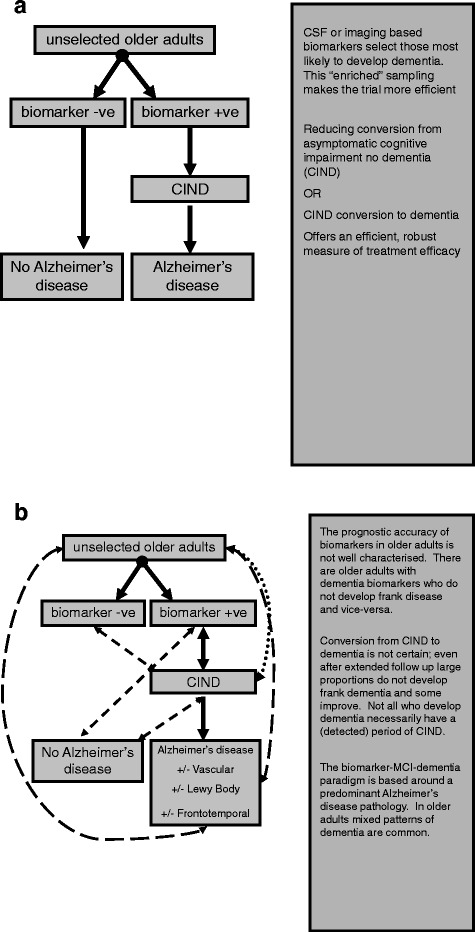
**The theory and reality of contemporary dementia research paradigms. (a)** An 'ideal' model, wherein older adults with early biomarker-detected changes of dementia can be selected and this cohort then progresses through a stage of 'cognitive impairment non-dementia' (CIND) with eventual overt dementia of a particular pathological subtype. **(b)** A more complex situation that is closer to the 'real world' of dementia research, wherein predictive accuracy of biomarkers is not 100% sensitive or specific, CIND to dementia conversion is neither predictable nor inevitable and the final syndrome of dementia is often a mix of underlying pathologies. CSF, cerebrospinal fluid; MCI, mild cognitive impairment.

Research has historically concerned dementia at the later stages, when definitive diagnosis can be made. Developments in imaging and molecular medicine are beginning to redefine our understanding of the natural history of dementia and this may in turn influence operational definitions of dementia states. The current understanding of (certain forms of) dementia progression describes neuropathological changes associated with development of dementia that may precede symptomatic disease by decades, cognitive change not yet sufficient to warrant a dementia label and then a state of overt dementia with progressive clinical severity [7] (Figure 1). For practical reasons we arbitrarily define these clinical stages, but the reality is of a continuum with no clear temporal delineation between stages. In fact some may reach a stage of cognitive impairment and not progress further, while others may even show reversion to 'normal' cognition. The ongoing debate as to where in the pathway research resources should be targeted is a reminder of our limited understanding of the dementia progression pathway.

### What about dementia subtypes?

The traditional approach to dementia has been to define a disease syndrome and then try to describe the underlying disease state. The diseases that cause dementia are defined in terms of classical autopsy-based neuropathology, yet we attempt to assign these labels in life through recognition of certain phenotypic patterns. The commonest cause of dementia in community dwelling older adults is Alzheimer’s disease (AD). AD research has accordingly tended to dominate the dementia landscape. Indeed many of the major scientific journals in dementia research have the term 'Alzheimer’s' in their title. To maintain scientific purity a focus of traditional dementia research has been around separating AD dementia from other dementia types.

Improvements in our understanding of later life dementia cause us to question the utility and validity of this rigid classification-based approach. Risk factors for AD and vascular dementia are shared [8] and the majority of dementia in older age is 'mixed' with varying degrees of vascular, amyloid and other pathologies [9]. In older adults correlation between clinical classification and the predominant neuropathology seen at autopsy is poor [10]. If the goal is to describe or target dementia at the population level, then strict classification-based inclusion/exclusion criteria may give data with limited external validity [11].

While at a population level treating dementia as a single entity may be appropriate, no-one would argue that we should stop trying to classify dementia completely. Indeed the increasing interest in stratified medicine would argue for greater pathological classification. We must also be mindful of not extrapolating research from a specific dementia group and applying the findings to the entire syndrome, the so-called Alzheimerisation of dementia. These apparently opposite approaches can exist together and there are examples of successful research paradigms where conditions have been assessed both as a syndrome and as individual disease groups. An example of this would be the field of stroke research; important bodies of work around small vessel disease stroke, intracerebral haemorrhage and cardioembolic stroke are all available, while large cohorts and trials have studied the stroke syndrome as a whole and yielded data that have informed practice.

### How do we quantify dementia in research?

**Figure 2:**
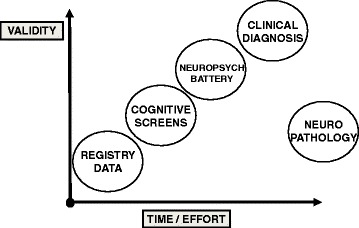
**Methods of assigning dementia diagnosis for clinical research.** Differing methods of assigning the dementia outcome are described in terms of the time and effort required to make the diagnosis (x-axis) and the external validity of that diagnosis (y-axis). The positions are illustrative only and designed to show the 'trade off' between effort and validity. In assigning validity we assume that expert clinical assessment is the reference standard; hence, neuropathological assessment requires substantial time/effort but validity is relatively low.

A consistent feature of dementia research is the inconsistency in how we measure the syndrome of interest. Dichotomous 'dementia' versus 'no dementia' outcomes have utility but can be methodologically inefficient with limited precision and responsiveness to change [12]. For describing dementia incidence or prevalence, there is a trade-off between the validity of the case ascertainment and the time and effort required. A gold standard of expert clinical diagnosis requires availability of expert assessors and access to appropriate investigations and ideally repeats assessment to document change over time. This approach is only possible at the individual patient or small scale study level. In contrast, using routinely recorded data, such as is held in primary care registries, can allow for relatively quick assessment of whole populations for those with a label of dementia. However, the resulting data will be less robust and in particular there are likely to be numerous 'false negatives' [13] (Figure 2). Even within a rubric of clinical diagnosis, there may be heterogeneity in the dementia assessment employed. A distinction could be made between the dementia diagnosis made in routine clinical practice and the diagnosis made as part of a research study, where the clinical study can potentially make use of expert adjudication panels and comprehensive ancillary investigations to give a robust diagnostic label while in routine care there may be more limited access to supplementary tests and any diagnostic label may be more nuanced.

**Figure 3:**
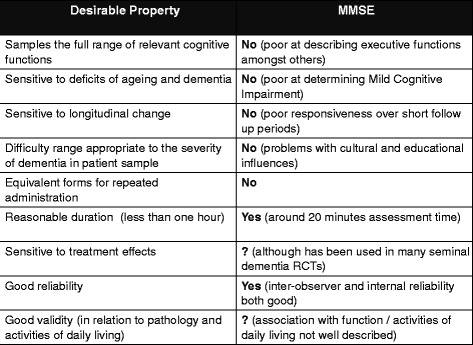
**Properties of cognitive assessment tools.** The first column describes the properties of an 'ideal' cognitive assessment tool (Ferris) and the second column describes a popular assessment tool (Folstein’s Mini-Mental State Examination; MMSE) against these desired properties. RCT, randomised controlled trial.

Various approaches to describing cognitive change as a quantitative variable have been described, all with vocal proponents [14]. Using some form of neuropsychological assessment to quantify cognitive impairment allows standardised assessment that does not necessarily require lengthy 'expert' input and gives a numerical output that can be used for analysis. An example of a prevalent assessment tool is Folstein’s Mini-Mental State Examination. This short, direct test of various cognitive domains has been used in seminal dementia studies but has a number of well documented limitations [15] (Figure 3).

Many other cognitive assessments are available with little guidance on the preferred tool(s). Even in a relatively niche area such as post-stroke cognitive assessment, around 300 different cognitive assessment tools have been used in research and 45 different tools used in clinical practice [16,17]. This substantial heterogeneity complicates attempts at meaningful comparisons across studies and effectively precludes pooled analyses of study results without substantial efforts to harmonize and co-calibrate the cognitive measures.

Choice of cognitive assessment tool for research should be guided by the properties of that tool and the purpose of the testing. Ferris and colleagues [18] described the ideal cognitive test, although this ideal is a theoretical construct and no existing tool is 'perfect' (Figure 3). There is a literature describing properties of cognitive assessment tools [19] and efforts to synthesize the evidence will hopefully bring greater clarity and consistency to the field [20].

Regulatory guidance for trials of pharmacological intervention in dementia suggests complementary approaches to cognitive assessment, using a performance-based measure of cognition and an independent clinician-rated measure of global disease severity [21]. The use of global measures was introduced to ensure that any change is clinically 'meaningful' assuming that important change will be apparent to the assessing clinician [22].

The dementia assessment is not only a cognitive assessment. Dementia is a state of cognitive decline sufficient to cause functional problems; thus, describing function is a critical component. As with cognition, functional assessment is challenging, particularly in an international, cross-cultural context. Further discussion of the challenges of functional assessment is given in the section on 'pre-dementia' states.

All the above assumes the biomedical perspective. Social and psychological sciences argue that these measures are overly reductionist and fail to capture the complex reality of dementia as experienced by those with the condition and their care-givers [23].

## Contemporary 'hot topics' in dementia research

### Dementia 'models' for research

Dementia is predominantly a disease of later life with a pathogenesis that may span decades. Thus, clinical studies may require protracted follow-up to assess outcomes of interest. One could argue that the focus should be unselected older adults. However, older adults bring inherent 'noise' in terms of comorbidity, frailty, polypharmacy and so on, as well as the problem of attrition due to death for non-dementia causes. Conversely, if we restrict studies to 'healthy' adults, then results have limited external validity. This dilemma is not unique to dementia; older adults with frailty or comorbidity are underrepresented in many studies and evidence-based guidelines may have limited relevance to 'real world' populations [24]. This challenge does not preclude meaningful research but emphasises a point that we will repeat in other sections of this review: we can only progress dementia research with cross-disciplinary collaboration that draws on expertise from geriatric medicine, neurology, psychiatry, psychology and other relevant fields. In the meantime more efficient methods of studying dementia are clinically and economically attractive. Unfortunately, dementia models currently available are problematic.

Researchers have used human phenotypic 'extremes' to study dementia - examples include the exclusively amyloid pathology that results from mutations in the *APP* and *PSEN1*/*2* genes and the monogenic disease CADASIL (cerebral autosomal dominant arteriopathy with subcortical infarcts and leukoencephalopathy) for vascular dementia [25]. Inherited dementias can give interesting information, but the relevance of these pure pathological states to sporadic dementia is at best limited. More common genetic variants can be employed to 'enrich' study samples (for example, the A4 study currently recruiting in North America). Genetic association studies have revealed a wealth of potential dementia risk markers but the effect of individual variants is often modest and it has been argued that a genotyping approach may be no more useful than a simple description of 'family history of dementia' [26].

Transgenic animal models of various pathological dementia subtypes have been described [27]. Translation of promising results from mouse to man has often yielded disappointing results [28]. Critical reviews of animal-based dementia work are available; for example, the Collaborative Approach to Meta-Analysis and Review of Animal Data from Experimental Studies (CAMARDES) group offer a critique of animal research in neurological disease and suggestions for improved conduct and reporting. They highlight recurring methodological limitations in animal research that mirror those seen in clinical studies, including non-blinding, lack of randomisation and publication bias [28]. Many of the problems in moving from bench to bedside relate to the external validity of the animal model employed. This is not solely a reflection of the increased complexity of the human brain compared with the mouse brain. As discussed, dementia usually happens as a result of mixed pathologies and in the context of a host of confounders that are difficult to simulate in the laboratory, including ageing, physical frailty and premorbid education.

Dementia is a progressive condition and an attractive approach is to use statistical models to describe trajectories of dementia-associated decline. Latent growth (or random effects) models are commonly used in dementia research. These approaches account for the auto-correlated nature of data generated from longitudinal studies and estimate average and individual trajectories using all available data [29]. Estimates of the heterogeneity of individual trajectories about the average trajectory are a key output of these models as they inform about individual differences in the evolution of disease. These models are highly flexible, as either parametric or non-parametric versions may be employed to describe non-standard trajectory shapes. However, there are limitations; although data from individuals with incomplete follow-up contribute to the model, missing observations are assumed to be random, an assumption implausible in the context of dementia where differential dropout and mortality operate. Secondly, results may be sensitive to features of the data and study design, such as ceiling and floor effects and separation of data collection waves. Thirdly, unless explicitly separated, model estimates may represent a compound of within and between individual sources of information [30]. Extensions of standard formulations of latent growth models (such as shared random effects models for missing data or Tobit models to account for ceiling/floor effects) have been proposed, although their application is not widespread [31].

**Figure 4:**
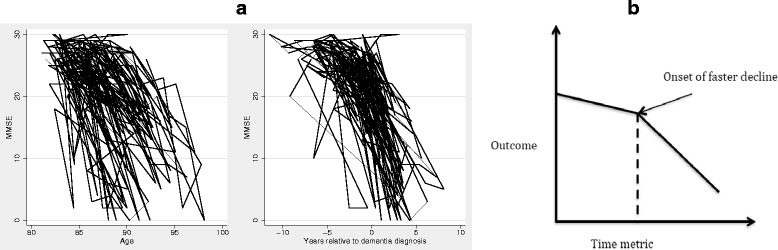
**Modelling cognitive trajectory. (a)** Comparing time to dementia and age to describe cognitive trajectories. Mini-Mental State Examination (MMSE) trajectories of a random sample of Origins of Variance in the Oldest-Old Twin Study participants plotted as a function of age and dementia diagnosis. As an illustration of how heterogeneity of trajectories is reduced when scores are modelled using a process-based approach, MMSE scores of a random sample of participants plotted as a function of age and time to dementia diagnosis are depicted [57]. **(b)** Graphical illustration of a change point model. A schematic representation of the typical change point model trajectory as assumed in the broken stick model [58]*.*

An important aspect of modelling dementia-related trajectories is the determination of the optimal time metric to best describe temporal changes of the process of interest. Intuitively, age may be considered as the natural metric to model change. A process-based approach where outcomes are modelled as a function of distance to the event that is most associated with the changes observed (for example, time to dementia diagnosis) has been shown to result in a reduction of residual variance estimates and better fitting models [32] (Figure 4a). Although process-based models result in better statistical fit, results may be hampered by the availability of accurate information about diagnosis. Change-point models (sometimes also called broken stick models) are a parameterisation of latent growth models that describe processes that occur in two phases with an abrupt change between them (Figure 4b). These models have been used in dementia research to estimate the onset of accelerated decline and are of particular interest to quantify change in rate of decline before and after diagnosis and identify risk factors that may differently affect the distinct phases of the disease and factors that may be associated with a delayed onset. Most applications of change point models have been estimated under the strong assumption of a common change point across individuals, although using Bayesian estimation techniques (as opposed to maximum likelihood estimation) random change point models have also been considered to estimate individual change points in preclinical dementia. Extensions to multivariate formulations of change point models have been employed to identify the temporal ordering of change [32] and models that assume a smooth, rather than an abrupt, transition have also been developed although not yet applied in dementia research [33].

### 'Pre-dementia' states and research

Accepting the current view of AD dementia progression, between asymptomatic pathological change and overt disease there may be a period of more subtle cognitive change not sufficient to meet diagnostic criteria for the dementia syndrome. This transitional state has been given many names, including mild cognitive impairment and cognitive impairment no dementia (CIND). DSM-V describes the functional decline of major neurocognitive disorder as ‘sufficient to interfere with independence’. Thus, the defining difference between CIND and established dementia is in functional ability, with social and occupational function preserved in CIND but impaired in dementia. Functional assessment in dementia is fraught with numerous challenges. Traditionally, we have used care-giver-based informant assessments. These tools are open to bias from care-givers’ mood and sense of burden and many commonly used scales only provide a snapshot of functional impairment. Functional assessment scales used in other areas of elderly care (for example, stroke research) may have some utility that could be applied in dementia research [34].

The differentiation of CIND from dementia states is crucial to research. Individuals with 'pre-dementia' are a target population for studies of novel prognostic and therapeutic interventions as this group theoretically offers a window of intervention opportunity before overt and irreversible cognitive change occurs. Recognising the therapeutic potential of early intervention there are currently around 124 registered trials of investigational pharmacological agents in mild cognitive impairment/CIND [35]. The US Food and Drug Administration mandates that conversion to dementia be used as study endpoint in treatment trials and in studies to validate biomarkers.

There are problems with the CIND conversion research paradigm. There is limited guidance on what constitutes CIND, particularly with regard to assessment of function. There is no consensus on which scales to use to measure functional ability or indeed what level of activity limitation is sufficient to merit a dementia label. As a result, CIND misclassification is prevalent in clinical trials with up to a third of participants enrolled as CIND misclassified and many already meeting criteria for dementia [36]. The erroneous inclusion of those who have early dementia into a CIND trial or failure to detect progression to functional impairment and dementia will substantially reduce trial power [37].

Temporal progression to established dementia is unpredictable and not inevitable (Figure 1). Annual rates for conversion of mild cognitive impairment to dementia of around 10% are quoted but meta-analysis suggests lower conversion over longer follow-up periods [38]. The study sampling frame may be relevant, with community recruited samples displaying much lower conversion rates than clinical samples [38]. 'Reversion' from CIND to states of normal cognition for age is also possible and further complicates the field as most models assume an irrevocability to dementia progression. The limitations of the clinical CIND definition led to proposals to enhance the process through use of biomarkers [3]. However, these proposals were lacking a strong empirical base [39] and early evidence suggests that biomarkers may not provide the hoped for improvement in accuracy [40].

### What is the role of biomarkers in research?

Biomarkers are defined as characteristics that can be objectively measured and used to evaluate biological processes (normal or pathological) as part of a diagnostic/prognostic evaluation or as indicator of response to intervention. Neuroimaging and tissue (mostly cerebrospinal fluid) based biomarkers have been described that may give an indication of early neuropathological change suggestive of future dementia [39,40]. In a relatively short time these biomarkers have been incorporated into diagnostic criteria and have been proposed as a novel method to improve patient selection for dementia research. An amyloid positron emission tomography ligand has been licensed on the basis of its utility in excluding a diagnosis of AD and the European Medicines Agency has supported the use of certain markers for studies of prodromal AD. There is a concern that biomarkers are increasingly used in routine clinical work, a practice not supported currently by any consensus clinical guidelines [41].

Two potential roles for biomarkers in dementia studies have been described. Using biomarker data as a surrogate outcome measure is intuitively attractive as the biomarker may offer early or more precise assessment of between-group differences. However, we must be mindful of high profile examples where positive trial data based on biomarker surrogates did not translate into meaningful clinical efficacy [42]. Biomarkers may also be used to define populations at risk of dementia and so 'enrich' study populations [3] as well as acting as intermediary phenotypes to decide on a drug’s continuation in a trial aimed at showing clinical benefit. This approach is at the core of the proposed Innovative Medicines Initiative-European Prevention of Alzheimer’s Dementia (IMI-EPAD) project.

We must be cautious in our enthusiasm, as there is still much we do not know regarding dementia biomarkers. The prognostic accuracy of biomarkers is substantially attenuated in older age; the proposed stepwise progression from biomarker change to dementia is not always apparent and time course is highly variable and there is not always a clear biological gradient between biomarker burden [41,43]. Existing statistical models employed to assess how changes in biomarkers impact on cognitive function are limited and need further development and the historic lack of standardisation in both sampling and analysis makes attempts at *post hoc* data harmonisation challenging. There are also ethical and feasibility issues. At present most biomarkers require detailed neuroimaging or invasive tissue sampling. Given the uncertainty around the 'meaning' of biomarkers, we need to be careful around consent and information disclosure in asymptomatic mid-life adults.

## Large scale studies

### Epidemiological studies in dementia?

The study of dementia epidemiology has presented several challenges, some specific to dementia and some common to other diseases.

A theme of dementia research has been looking to define modifiable risk factors that may in turn prove to be targets for intervention. Dementia, like many common non-communicable diseases, is the end result of a complex interplay of genetic, lifestyle, clinical and environmental factors. Given the multifactorial nature of dementia, the strength of association for any one risk factor is likely to be modest and very large populations may be required to detect meaningful signals [44]. Some have argued that it is overly simplistic to suppose that a single factor will be responsible for a substantial proportion of older age cognitive decline and researchers should focus on identifying groups of interrelated/interacting factors that are potentially causal or protective. A better understanding of the frequency of risk factors in the community should influence public health policy [45].

If we accept the current model of dementia with its long latent period, it is difficult to define an ideal time to begin study. Assessing late in the disease process may miss opportunities, while assessing very early will require follow-up periods that are not feasible using current study methods. There are few large prospective studies that offer follow-up from mid-life or younger though several have recently initiated. Studying associations at various time points in the dementia pathway is important as the role of certain 'risk factors' may change as disease progresses [46]. Novel programmes seek to develop complex mid-life models associating risks with disease manifestation and longer term clinical and cognitive outcomes [46,47]. This focus on mid-life risk and dementia was highlighted in the recent Blackfriar’s Consensus on Promoting Brain Health [48].

Dementia and other non-communicable diseases share many risk factors, not least of which is older age itself. This may cause problems of competing risk where those with potential to develop dementia die of other diseases before cognitive decline is manifest. Another challenge of studying a multifactorial disease with a long 'latent' period is that the environmental context may change over the period of study. As an example, the last few decades have seen substantial changes in population levels of education. Education can impact on dementia expression and current dementia incidence and prevalence figures for older adults (based on educational and social norms of the first half of the 20th century) may not extrapolate to the future [49].

As there is a ready supply of 'substrate', many dementia studies have been conducted in the memory clinics or wards of academic centres with a research interest in dementia. The external validity of these cohorts is questionable. For longitudinal studies where patients are recruited in specialist centres there needs to be robust links with community and primary care for follow-up. Recent European initiatives have sought to ‘re-purpose’ observational cohorts and develop community-based registers of patients. The creation of such ‘readiness cohorts’ for trials is one of the primary activities in the creation of the UK Dementia Platform [50].

The ideal would be to study dementia using a representative sample and a life course perspective. This is potentially challenging and costly but not impossible. There are examples of high quality, longer term prospective studies that are helping improve our understanding of dementia - the Medical Research Council Cognitive Function and Ageing Study is an exemplar [48]. Such approaches require considerable investment to set up and run. To maximise the potential return on this investment there needs to be early consideration of data storage and data linkage (mindful of data sharing and privacy concerns) to external data sources such as electronic patient records as well as to other relevant cohort databases, an approach being taken in the Innovative Medicines Initiative European Medical Information Framework (IMI EMIF-AD) project [51].

### Clinical trials in dementia

The ultimate goal of much of the research activity in dementia is to develop interventions for 'treatment' or even 'cure'. The classical bench to bedside paradigm has been disappointing in dementia. There is a long list of putative dementia treatment compounds with favourable pre-clinical and early phase trial data that have been neutral or even potentially harmful when assessed in phase III studies [28].

The 'gold standard' for testing efficacy is the prospective, blinded, randomised controlled trial (RCT). Regulatory authorities took an early interest in dementia RCTs and proposed a framework for assessment that is still used today. Thus, dementia treatment RCTs have historically been fairly robust, but there may still be scope to improve. Indeed there is a plausible argument that the traditional single intervention RCT paradigm is not suited to a complex multifactorial condition such as dementia [46]. An issue specific to studies looking at later stages of dementia is around consent to randomisation/intervention. The complexity of contemporary dementia research interventions can make informed consent challenging even for patients with no cognitive issues. The consent, recruitment and retention of patients with progressive cognitive decline is problematic and further complicated by country-specific differences in legislation - for example, around proxy-based consent.

Large scale, international RCTs are an expensive endeavour, with number of participants recruited being a major factor in total cost. Expectations of treatment effects from previous RCTs in dementia may have been overly optimistic with possibility of type II statistical error. Given the prevalence and disability associated with dementia, even modest treatment effects many still be important at a population level [26]. Data from other neurological diseases suggest that optimising basic aspects of study process, such as improving classification of outcomes, can have a substantial impact on required sample size and ultimately cost of the study [37].

As with the 'life course' epidemiology studies discussed previously, the cost of multicentre RCTs must be balanced by efforts to maximise added value. The robust phenotyping and outcomes assessment of RCTs provides a data resource that may be used for testing future novel hypothesis. Collating anonymised, individual patient level data across several such RCTs in a single resource designed for future research has been demonstrated to have feasibility in the field of cerebrovascular medicine [52,53]. In both RCTs and prospective cohorts, collecting baseline and follow-up samples to create a 'biobank' of tissue, imaging and genetic materials as well as clinical outcome data with broad consent that allows for future research and sharing will increase the potential research utility beyond the primary aims of the original study. If participants give consent for neuropathology, the research potential increases further still. In all of this, consideration must be made to 'future-proofing' the data so that data are standardised to allow harmonization with data from other resources.

Data from 'non-memory' RCTs can be used to progress the dementia research agenda. High profile examples from North America include the Framingham Heart Study and the Honolulu Asia Aging Study, both of which added cognition-based analyses to the existing cardiovascular data [54]. Contemporaneous dementia assessment of a population with detailed historical phenotyping has allowed exploration of mid-life risk factors with later life cognitive decline.

Greater harmonisation and a culture of sharing experience and best practice in dementia treatment and prevention trials may help progress the dementia research agenda with specific consensus statements appearing [55] and the creation of conduct and reporting guidance specific to dementia studies [56].

## Conclusion

A dementia 'cure' remains elusive. One could speculate that problems with trial design, endpoint definitions and analysis may be contributory. However, we should avoid research nihilism; there has been substantial advance in our understanding of dementia and as we develop new techniques and technologies there is cause for cautious optimism. Based on the discussion in this review we offer some pointers for future dementia research initiatives.

Studies of dementia should recognise the potential disconnection between a pure pathological state and the clinical syndrome of late life dementia. We must be mindful of extrapolating results for 'focussed' samples to an unselected all-cause dementia population.

Inconsistency in choice and reporting of outcome measures is problematic. Based on evidence of test properties, we should look to build a core set of standardised outcomes that can be supplemented by study-specific measures. Where there is guidance on best practice in reporting studies we should follow this.

Although advanced statistical models have been developed and applied to describe trajectories of cognitive change, model assumptions and features of the data and study designs need to be accounted for when implementing these models in dementia research. Further collaboration between methodologists and clinicians should be encouraged for the development of models that fully consider the complexities of dementia studies.

Biomarkers potentially have an important role in patient selection or as study outcome; however, the relevance and utility of these tests in an unselected older adult cohort is still to be described. Innovative study designs will be required to capture the complexity of dementia-related declines/biomarker changes and lifestyle factors associated with these changes.

The complexity of dementia requires an international collaborative approach, and examples of such efforts are available [46,54]. This will be particularly important to allow adequately powered phase III trials of prevention or intervention.

### Note

This article is part of a series on *The impact of acute and chronic medical disorders on accelerated cognitive decline’*, edited by Carol Brayne and Daniel Davis. Other articles in this series can be found at http://alres.com/series/medicaldisorders.

## Abbreviations

AD: Alzheimer’s disease
CIND: Cognitive impairment no dementia
DSM: American Psychiatric Association Diagnostic and Statistical Manual of Mental Disorders
ICD: World Health Organization International Classification of Disease
RCT: Randomised controlled trial
